# Use of the speckle imaging sub-pixel correlation analysis in revealing a mechanism of microbial colony growth

**DOI:** 10.1038/s41598-023-29809-0

**Published:** 2023-02-14

**Authors:** Ilya Balmages, Janis Liepins, Ernests Tomass Auzins, Dmitrijs Bliznuks, Edgars Baranovics, Ilze Lihacova, Alexey Lihachev

**Affiliations:** 1 Faculty of Computer Science and Information Technology, Department of Computer Control and Computer Networks, Riga Technical University, Zunda Krastmala 10, LV-1048 Riga, Latvia; 2 Laboratorija Auctoritas Ltd, Čiekurkalna 1. linija 11, Riga, LV-1026 Latvia; 3 Institute of Microbiology and Biotechnology, University of Latvia, Jelgavas str. 1, Riga, LV-1004 Latvia; 4 Institute of Atomic Physics and Spectroscopy, University of Latvia, Jelgavas str. 3, Riga, LV-1004 Latvia

**Keywords:** Optics and photonics, Applied optics, Optical techniques, Biological techniques, Imaging, Microbiology, Applied microbiology

## Abstract

The microbial colony growth is driven by the activity of the cells located on the edges of the colony. However, this process is not visible unless specific staining or cross-sectioning of the colony is done. Speckle imaging technology is a non-invasive method that allows visualization of the zones of increased microbial activity within the colony. In this study, the laser speckle imaging technique was used to record the growth of the microbial colonies. This method was tested on three different microorganisms: *Vibrio natriegens*, *Escherichia coli,* and *Staphylococcus aureus*. The results showed that the speckle analysis system is not only able to record the growth of the microbial colony but also to visualize the microbial growth activity in different parts of the colony. The developed speckle imaging technique visualizes the zone of “the highest microbial activity” migrating from the center to the periphery of the colony. The results confirm the accuracy of the previous models of colony growth and provide algorithms for analysis of microbial activity within the colony.

## Introduction

The growth of colony size is used as a criterion for characterizing the microbial activity on agar media. Colony size growth is usually monitored by recording live images at different times and comparing the sizes of the colonies. Adopting this imaging technique, images are obtained by high-resolution cameras or flatbed scanners^1,2^*.* Critical detection time, colony size, growth rate, and maximum colony size are the parameters, which may be quantified using the existing live imaging methods.

There are several models describing colony growth^3–5^. In many models, microbial colony growth is described to be uneven—colony growth depends on the active zones of the microbes which, in their turn, depend on nutrient supply from the media. For example, microbial growth on the edge of the colony is more active than in its center due to the constant supply of nutrients from the surrounding media or the constant push of the cells on the lateral ones. The microscopic structure of the colony supports many of these presumptions. The cells in the center of the yeast colony stop proliferating and lose viability while the cells on the edge of the same colony are alive and continue growing (proliferating)^6^. The differences in microbial activity in different parts of the colony cannot be accurately visualized with the existing live imaging methods. Currently, different microbial activity within colonies can be explored only with invasive procedures—colony cross-sectioning and viability staining^6^.

An interference pattern created by the coherent light reflected or scattered from different parts of the illuminated surface is a laser speckle. If an object that is irradiated with laser speckles does not change the scattering properties over time, the speckle image would not change either. If the scatterers move spontaneously (e.g., Brownian motion), some speckles begin to change (shape, intensity). The change in the scattering properties of objects creates a time‐varying speckle. That is, external events or influences, as well as processes occurring directly on the measured surface, can lead to changes in the speckle images^7^. Regarding this phenomenon, the laser speckle imaging technique has been proved useful in monitoring microbial activity in optically inhomogeneous media by analyzing time-varying laser speckle patterns.

The laser speckle imaging technique is an emerging technology for medical and food safety analyses. It allows for early detection of microbial growth^8^, identification of fungal infection in apple fruits^9^, and measuring peripheral blood flow in sclerosis patients^10^.

In this study, the speckle imaging technique was used to record the growth and structural features of the microbial colony. The results showed that the developed speckle analysis system is capable not only to record the growth of a microbial colony but also to visualize microbial growth activity in the different parts of the colony. The speckle imaging reveals that colony growth is driven by cell proliferation on its edges rather than its center. The results confirm the accuracy of the previous models of colony growth and provide algorithms for microbial activity analyses within the colony.

## Materials and methods

### Microbial strains and cultivation conditions

The following microbial strains were used in the experiments: *Escherichia coli* ATCC^®^ 8739 (*E. coli*), *Vibrio natriegens* DSM 759 (*V. natriegens*), and *Staphylococcus aureus* ATCC^®^ 6538P (*S. aureus*). Bacterial cultures were maintained on agar plates. *E. coli* and *S. aureus* were maintained on LB agar media consisting of Bacto Peptone 10 g/l, yeast extract 5 g/l, agar 20 g/l (all Biolife, Italy), and NaCl 5 g/l (Sigma, Germany). *V. natriegens* was maintained at room temperature on agarized NB salt broth: Difco Nutrient Broth 8 g/l, NaCl 15 g/l, as suggested by Weinstock et al*.*^11^.

All microbial species were subcultured from a single colony in the respective liquid media and cultivated at + 30 °C overnight. Petri plates were inoculated with serially diluted bacterial culture to yield 20–200 colonies per plate. Petri plates with inoculated bacteria were placed at + 30 °C in an incubator (Herracell 240-i, Thermo Scientific). Colony growth was monitored in parallel by 635 nm laser illumination (see description in "Experimental system for capturing the laser speckle images and images under broadband illumination") and by flatbed document scanner (CanoScan 4400f, Canon). Automated plate scanning after every 15- or 30-min interval was ensured by running the Auto Clicker app. The resolution of the pictures was 600 DPI.

### Experimental system for capturing the laser speckle images and images under broadband illumination

**Figure 1:**
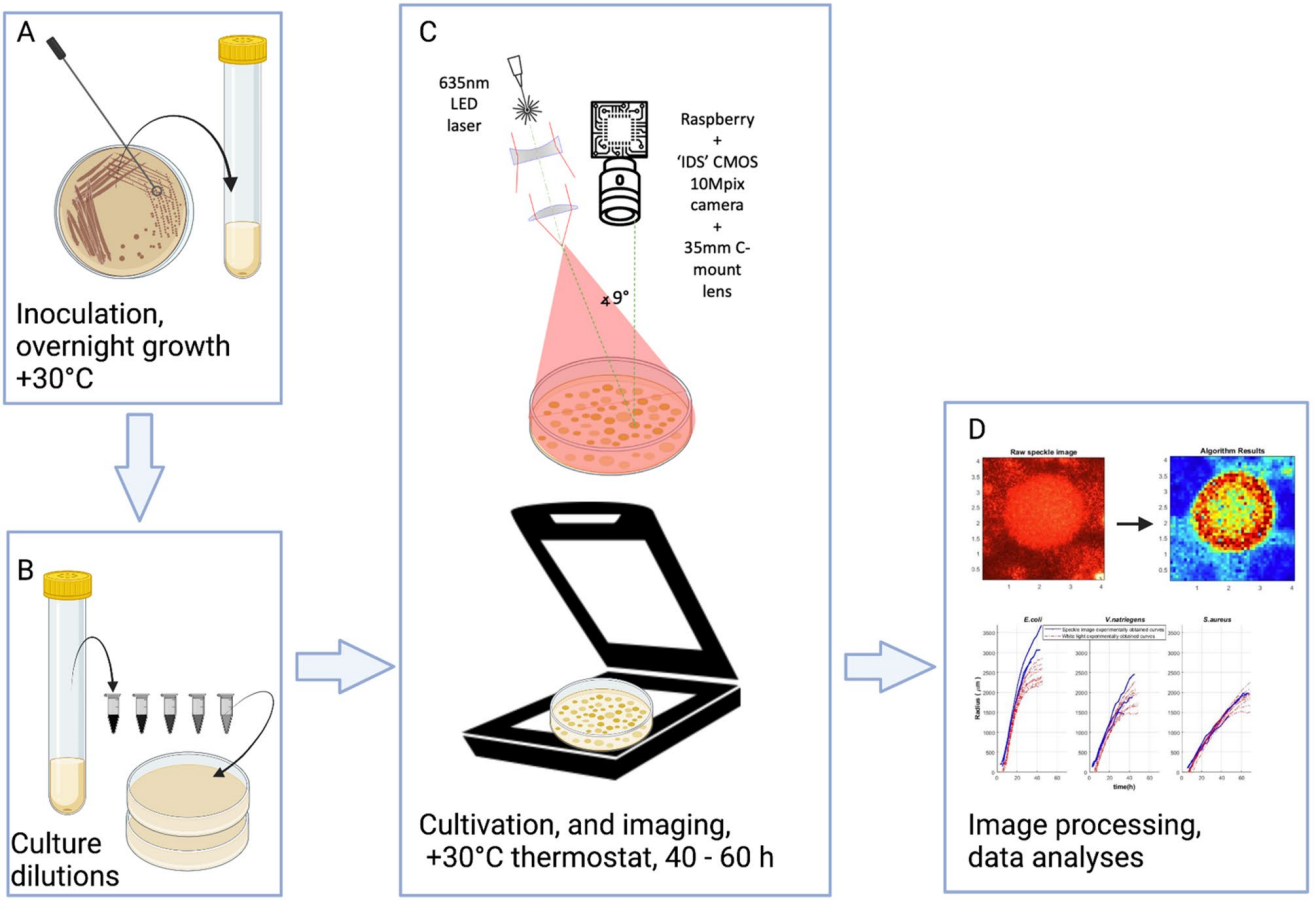
Experiment workflow for speckle image capturing during bacteria growth. (**A**) Single colony inoculation to a LB media and overnight cultivation (+ 30 °C). (**B**) Dilution and inoculation of overnight culture on agarised LB media. (**C**) Colony growth on agar plates in thermostat + 30 °C with speckle image capturing under 635 nm laser and flatbed scanner. D Image processing and data analysis (MATLAB environment): detection of colony size, growth speed, filtration of speckle signal, identification of speckle migration, etc.

The experimental laser speckle imaging system was assembled for capturing macro scale images under white light LED and red laser illumination. The system consists of a multimode laser source, white LED light source, 35 mm C-mount lens @F18, optical attenuator, a testing agar plate (with inoculated bacteria), and a CMOS camera (Fig. 1). Laser speckles were generated by a linearly polarized 635 nm multimode diode pumped solid state laser (output power up to 300 mW) with a coherence length of 30 cm. The optical attenuator was used to achieve the optimal exposure for image capturing and to avoid heating effects of the illuminated plate, enabling 3–5 mW/cm^2^ power density of the scattered laser light on the entire agar plate surface. The diameter of the laser beam on the surface was greater than 9 cm providing even illumination of the entire standard Petri plate. The main components of the system are presented in Fig. 1. The speckle images were captured by a CMOS camera at 30-s intervals for experiments with different durations (25–70 h). Exposure time was set to 1 s; it was chosen according to laser illumination and lens diaphragm. Parameters of the optical setup including camera resolution, lens diaphragm, camera distance to Petri plate, and the resulting region of interest were chosen to achieve adequate spatial resolution for detecting laser speckles. Lens magnification was set to 0.2. Diaphragm value of F18 was chosen as the optimal balance between image sharpness, speckle size, and the required exposure. That results of spatial resolution of 9 μm and speckle size of 33 μm.

### Algorithm of the speckle image conversion into time signals

In the previous studies, the authors described sensitive correlation subpixel analysis, which can help detect bacterial activity and its effects within a colony^8,12^. The entire field of the experiment (Petri dish) was divided into small sections of N × N pixels. The steps below were realized for each section separately. A two-dimensional normalized cross-correlation was performed between consecutive images of N × N pixels from the beginning to the end of the experiment^13^:

1 $$NCC\left(u,v\right)=\hspace{0.33em}\frac{{\sum }_{x}{\sum }_{y}\left(\left(a\left(x,y\right)-\overline{a}\right)*\left(b\left(x-u,y-v\right)-\overline{b}\right)\right)}{\sqrt{{\sum }_{x}{\sum }_{y}(a(x,y)-\overline{a}{)}^{2}*{\sum }_{x}{\sum }_{y}(b(x-u,y-v)-\overline{b}{)}^{2}}},$$

where a(x,y) and b(x,y) are two adjacent frames in the sequence, $\overline{a}$ and $\overline{b}$ are the average values of these two frames, u and v are spatial displacements between frames a(x,y) and b(x,y) towards x and y, respectively.

It was necessary to find out the exact shift of the cross-correlation peak. This value characterizes the changes that occur between consecutive NxN pixels images:

2 $$\left( {\hat{u},\hat{v}} \right) = \mathop {\arg \,\max }\limits_{u,v} (NCC(u,v)).$$

To find a more accurate value of the offset, the parabolic interpolation was performed around the peak of the cross-correlation function^14^:

3 $${\hat{\delta }}_{x}=-\frac{{b}_{u}}{2{a}_{u}}=\frac{NCC(\hat{u}-1,\hat{v})-NCC(\hat{u}+1,\hat{v})}{2\left(NCC(\hat{u}-1,\hat{v})-2NCC(\hat{u},\hat{v})+NCC(\hat{u}+1,\hat{v})\right)},$$

where ${a}_{u}$ and ${b}_{u}$ are the parabolic coefficients.

The offsets obtained between each pair of adjacent NxN pixels images were accumulated from the start to the end of the experiment:

4 $$sig\left[n\right]={\sum }_{i=1}^{n}\hat{\delta }\left[i\right].$$

Running the described algorithm for consecutive N × N pixels images for the entire sequence creates a “time signal”.

This algorithm was implemented for all N × N pixel sections in the entire field of the experiment. Thus, a two-dimensional array from the “time signals”, where time is the third dimension, was obtained in place of the considered field of the experiment.

**Figure 2:**
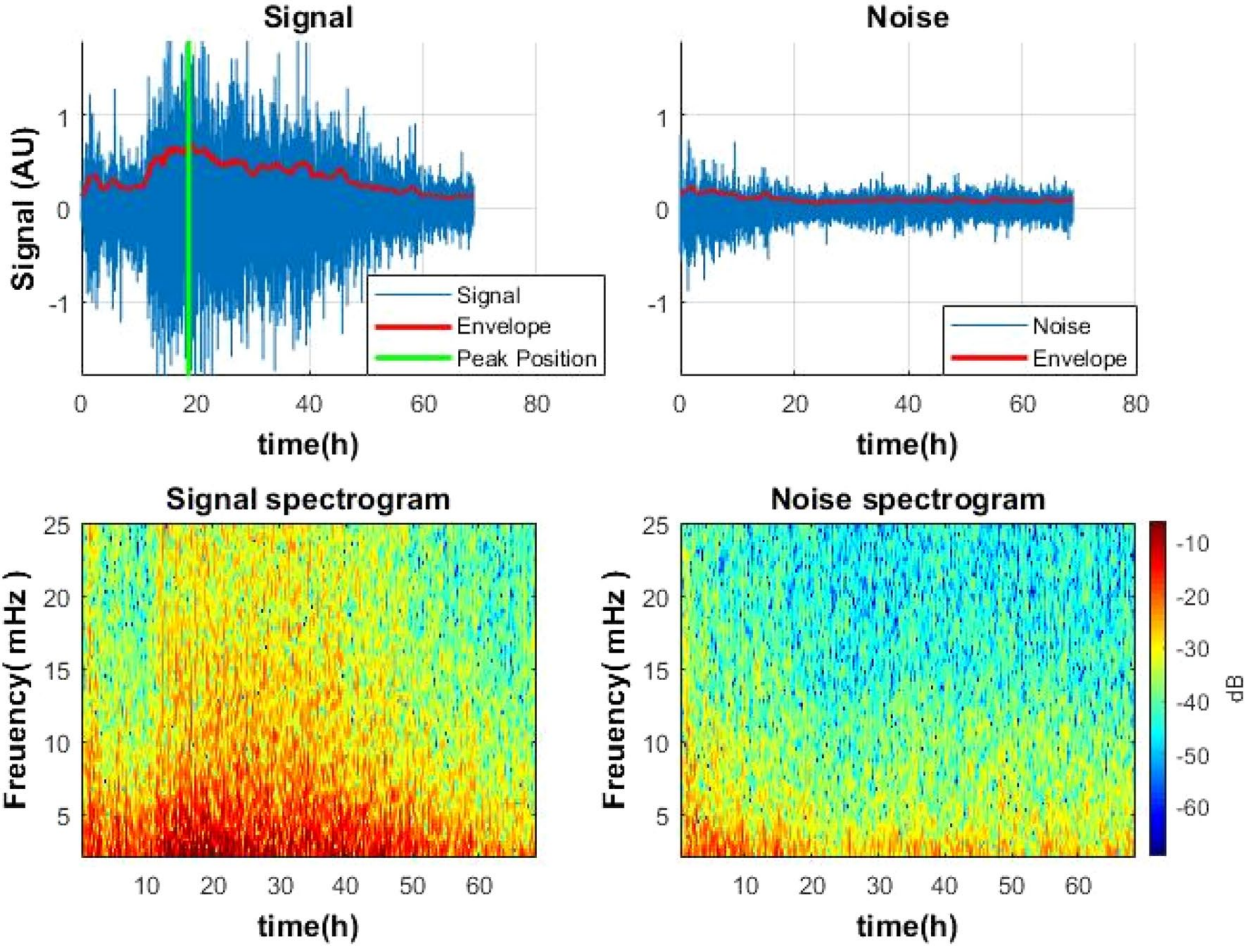
Top left: The signal obtained using the algorithm (blue) and its envelope (red). (Signal from the colony center). The green line indicates the time of the maximum activity. The figure shows a signal from a *S. aureus* colony. Similar speckle signal pattern was observed also for *E. coli* and *V. natriegens*. Bottom left: A spectrogram is a representation of a signal on a time–frequency domain using a short-time Fourier transform (STFT), allowing simultaneously to observe the behavior of the signal and noise in time and frequency. Right side: the noise (out of the colony) obtained using the algorithm (top) and noise spectrogram (bottom).

To find the local extrema and to avoid the influence of local transient spikes, it is expedient to smooth the signal^15^. The signal envelope function was used (Fig. 2). A moving root-mean-square technique or another similar algorithm can be used for this purpose:

5 $$Env\left[n\right]=\sqrt{\frac{1}{N}{\sum }_{k=n-N+1}^{n}sig[k{]}^{2}},$$

where N is the length of the window, n is the current sample, k is the index running inside the window. Accordingly, N—the length of the window—is responsible for the degree of signal smoothing. To avoid outliers when performing the RMS technique, the extreme values can be truncated, as it is done adopting the truncated mean technique^16^.

On Fig. 2, it is also possible to compare the signal (inside the colony) and the noise (outside of the colony) both in time and frequency domains. The signal is much higher than the noise level.

## Results

### Dependence of laser speckle signal on microbial colony growth

**Figure 3:**
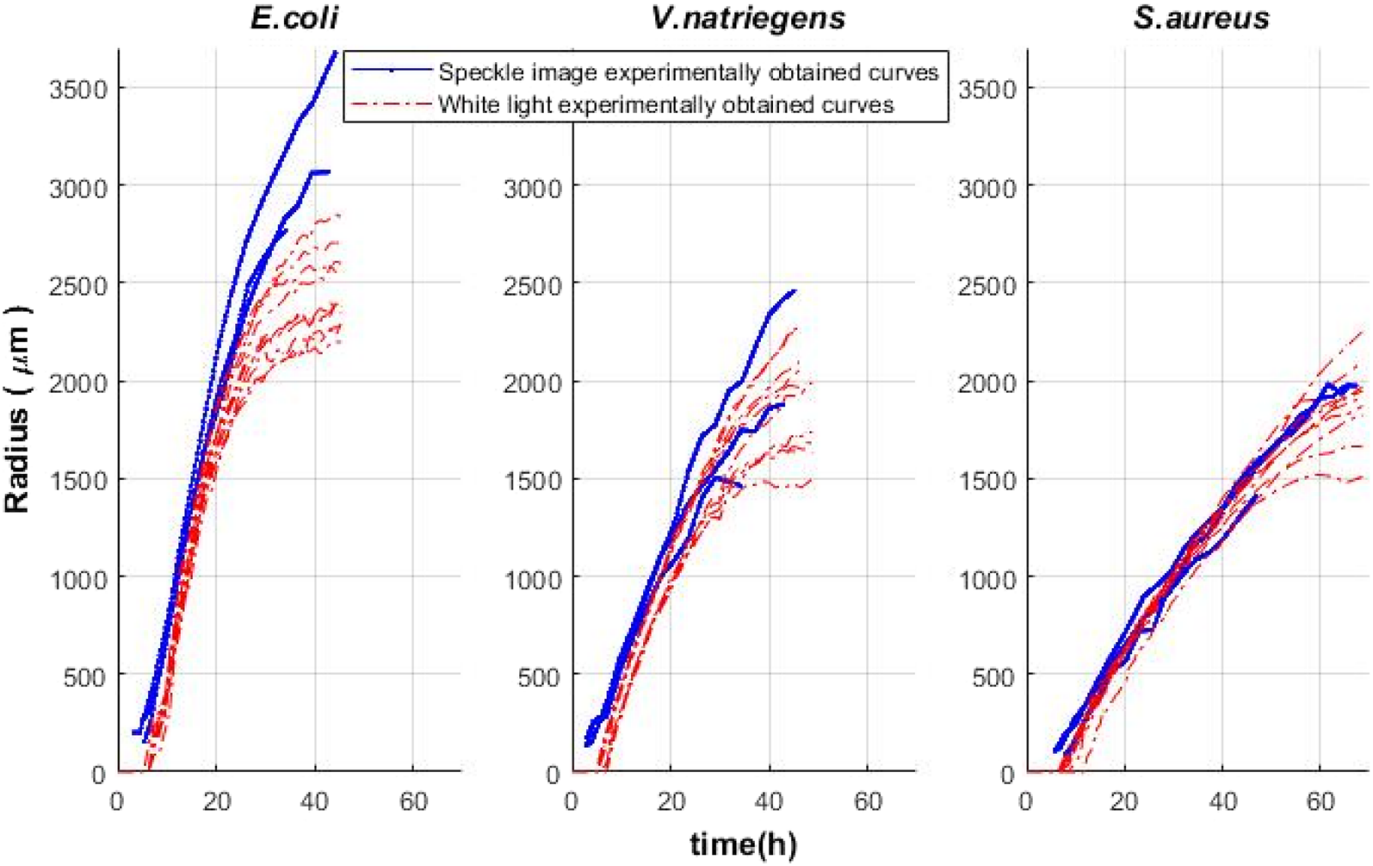
Dynamics of colony growth radius over time. The colony radiuses of *E. coli, V. natriegens* and *S. aureus* were measured by laser speckle image analysis (blue) or considering images taken by flatbed scanner (Canon 4400, 600 DPI) (red).

First, it was tested whether the established laser speckle imaging system did not affect the growth of microbial colonies. To verify this, the colony growth radius estimated by the laser speckle system was compared to the results obtained using the reference method—a well-recognized flatbed scanner imaging system. For this purpose, microorganisms were subcultured from a single colony overnight, serially diluted, then either inoculated on a Petri plate to observe colony formations from a single cell (approx. 20–200 colonies per plate) or by applying spots from serial dilutions on the plates and thus obtaining macrocolonies. Micro and macro colonies on an agar medium were grown in the same incubator in parallel under a speckle imaging system and on the flatbed scanner (Canon Canoscan4400r). The growth of *V. Natriegens*, *E. coli,* and *S. aureus* colonies was recorded, and their speckle patterns were analyzed using a sub-pixel correlation algorithm (described in "Algorithm of the speckle image conversion into time signals"). The results are presented in Fig. 3.

Comparing the growth of the tested colonies, very similar results were obtained with both systems. Therefore, it may be concluded that the established laser speckle system does not affect the growth of microbial colonies (see Fig. 3). We compared the radial growth rate of microbial colonies grown under speckle imaging device and within a flatbed scanner and radial growth rate did not differ significantly (t test, p > 0.05), see Supplementary Table 1.

**Figure 4:**
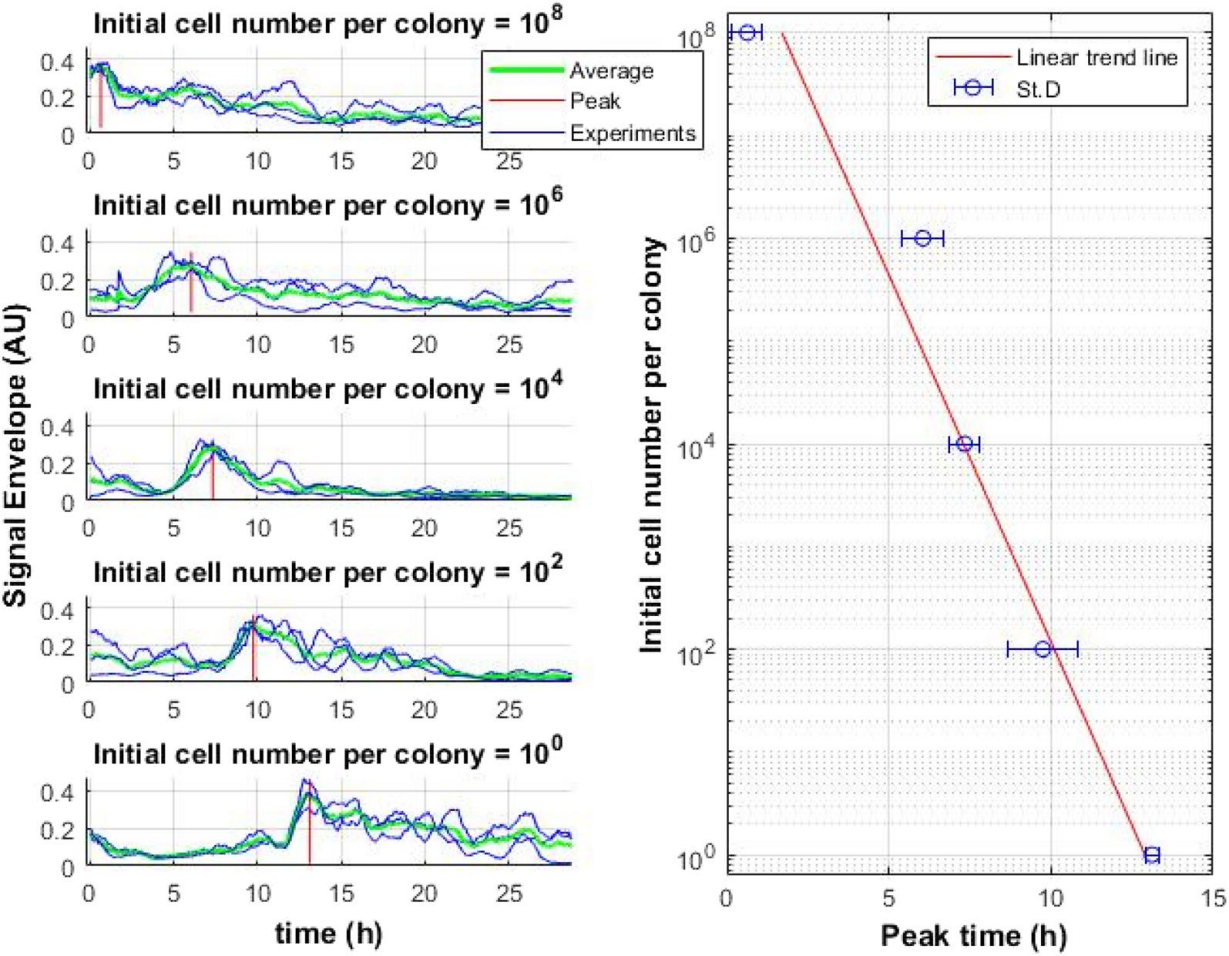
Dependance of the time of the maximum signal value (red lines on the graph on the left and blue circles on the graph on the right) obtained by the laser imaging system on the initial number of *E. coli* cells in the colony.

In addition, it was analyzed whether the speckle signal of the growing colony correlates with the number of cells in the colony. The time of the maximum signal value of the laser speckle images was compared with the initial cell number within the macrocolony (see Fig. 4). The time of the maximum signal value is defined as the time when the signal activity reaches its maximum. Overnight *E. coli* culture was serially diluted and inoculated on the LB agar media as macrocolonies (the volume of each inoculum was 5 μL). Macrocolony growth was recorded using a laser speckle imaging system. The time of the maximum signal value for each macrocolony was determined and plotted against the initial cell number per macrocolony. Signal peak time mean values and standard deviation of the three independent inoculation series were calculated (see Fig. 4).

The first results demonstrated that colony growth within a speckle imaging system was the same as in a flatbed scanner. Slight differences between them arise from the individual growth of the colony, which can be affected by the localization of the colony within the plate or the distance to the nearest neighbors^17^. Meanwhile, the time of the maximum signal value of the speckle signal depends on the initial cell number within the colony. If the initial number of cells in the colony is large, less time is required to reach the maximum speckle signal. On the other hand, if the initial number of cells is small, it will take longer to reach the maximum speckle signal value. Even though colony growth continues for more than 20 h (see Fig. 4 for *E. coli*), the maximum of the speckle signal is reached once and it never “recovers”.

### Sub-pixel correlation analysis for determining spatiotemporal activity patterns during colony growth

#### Detection of high microbial activity zones using the laser speckle imaging system

**Figure 5:**
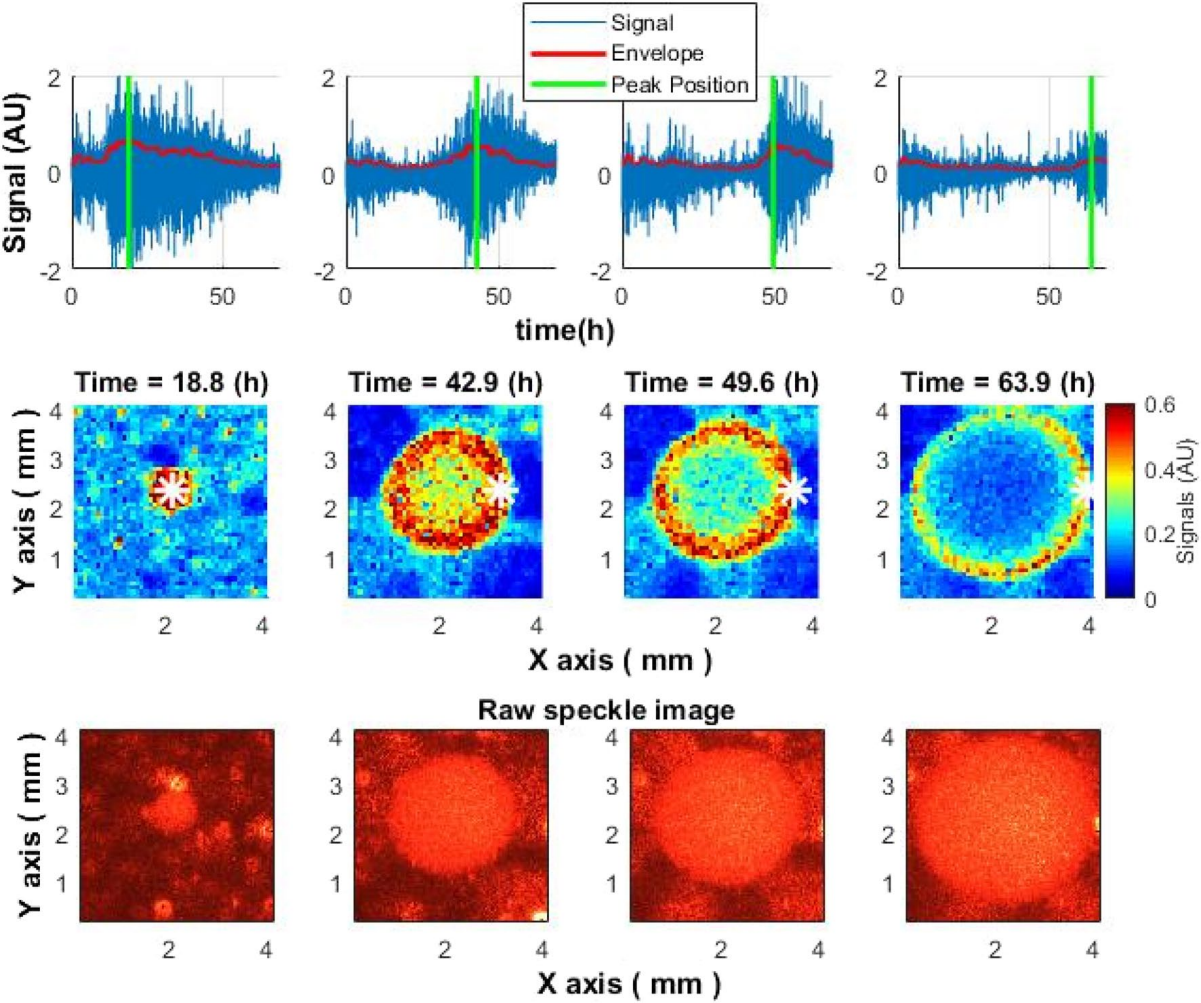
Spatiotemporal distribution of the speckle signal across *S. aureus* colony. Upper row: signal dynamics over time at different points within the colony (white star in the middle graphs). The green line marks the maximum activity, after which the signal decreases. Middle row: changes in the activity during colony growth. Bottom row: Raw speckle images.

**Figure 6:**
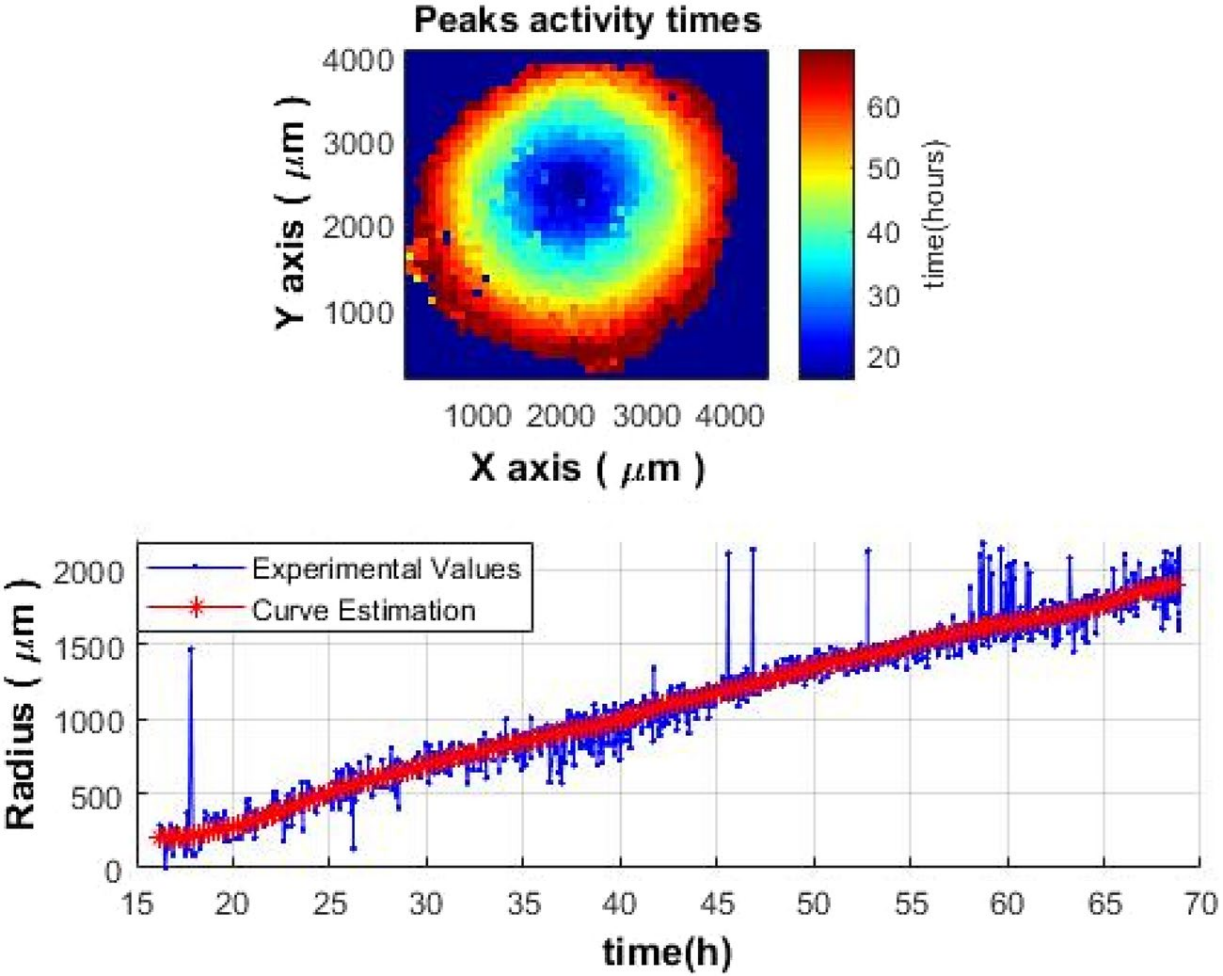
The time of colony peak activity (the start of the decrease in activity) during the growth of *S. aureus* colony. Top image—a two-dimensional spatiotemporal image; bottom image—the peak activity as a function of time. Similar spatiotemporal behavior of the signal was observed also within colonies of *E. coli* and *V. natriegens.*

The signal obtained by sub-pixel correlation of laser speckle images, which characterizes bacterial activity, increased inside the colony, reached its maximum, and then decreased. The signal strength had a remarkable spatiotemporal distribution. Different parts of the colony had different maximum signal peak times. The maximum activity peak was initially observed at the center of the colony. Over time, activity “wave” migrated from the center to the edges of the colony (see Figs. 5 and 6). This spatiotemporal behavior of the speckle signal was observed within all tested microbial colonies: *S. aureus*, *E. coli* and *V. natriegens*. Even though in depth analyses on the structure of the speckle signal migration was done on several colonies from three different microbial species (*V. natriegens*, *E. coli* and *S. aureus*), signal migration away from the center to the edge of the colony is feature of every and each microbial colony. In Supplementary Fig. S1 shows an example of part of a Petri dish with about 25–30 colonies. Each of the colonies after subpixel correlation analysis shows the formation of the activity ring effect described above.

It is possible to obtain an image that illustrates the beginning of the decrease in activity throughout the colony (see Fig. 6, top image) by choosing the times of the start of the decrease over the entire area of the colony and placing them on a two-dimensional spatial map with the corresponding coordinates.

The high activity “ring” is formed around the center and then it migrates away together with the front of the growing colony. The color shows the time (in hours) when the activity started to decrease: the blue color (in the center) represents the earliest, red color represents the latest (edge of the colony).

The location of the first decrease on the map can be assumed as the starting point for the growth of a microbial colony. Knowing the coordinates of this starting point or center, it is possible to obtain a curve of the colony growth as a function of time. However, since the growth of the colony depends on various factors (the availability of oxygen and nutrients, Brownian movement, push and/or pull from the neighboring cells within the colony, asynchrony of the cell growth and proliferation within the colony, etc.) the resulting curve will have a certain scatter of values (Fig. 6, bottom graph). Therefore, a moving median filter was applied to smooth curve data^15^:

6 $$Dsmooth\left(n\right)=median\left[D\left(n-N+1\right),D\left(n-N+2\right),...,D\left(n-1\right),D\left(n\right)\right],$$

where N is the length of the window, n is the current sample. The median is the number that is situated in the middle of a set of numbers when sorted in ascending order. That is, half of the elements in the set of numbers are not less than the median, and the other half is not greater than the median:

7 $$median(x)=\left\{\begin{array}{c}x\left(\frac{N+1}{2}\right)\quad if\,N\hspace{0.33em}is\hspace{0.33em}Odd\\ \frac{x\left(\frac{N}{2}\right)+x\left(\frac{N}{2}+1\right)}{2}\hspace{0.33em}\quad otherwise.\end{array}\right.$$

This method was applied for the following reasons: (1) It does not try to fit the curve into a specific shape: a straight line, or a parabola, etc. (2) Unlike a moving average, it does not take outliers into account.

### Analysis of activity zones for different bacterial colonies in the time–space domain

**Figure 7:**
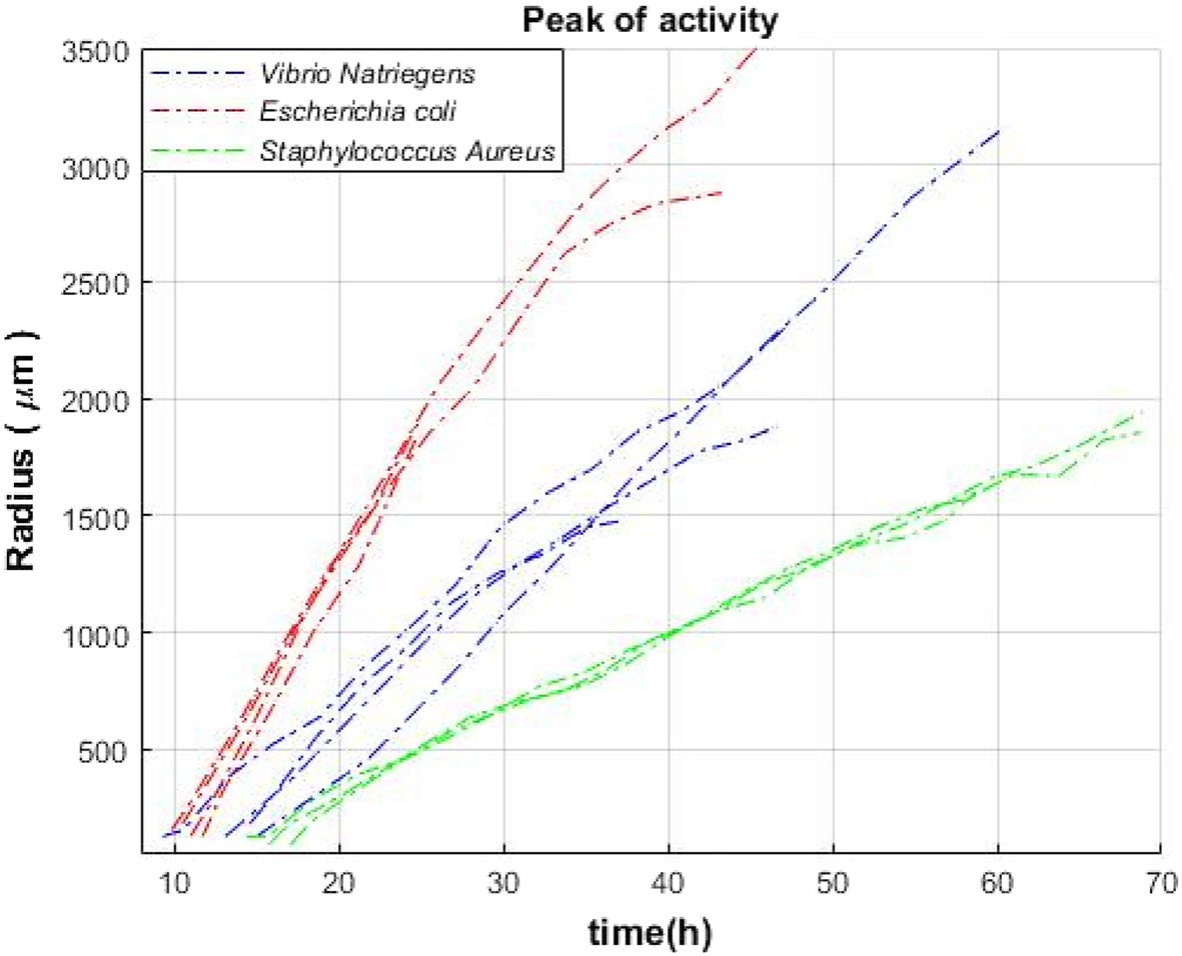
Colony peak activity curves as a function of time for 3–4 V*. natriegens* (blue), *E. coli* (red), and *S. aureus* (green) colonies.

The “ring” effect was obtained for all three types of the tested bacteria. The growth rate was the main difference that was observed (Fig. 7).

**Figure 8:**
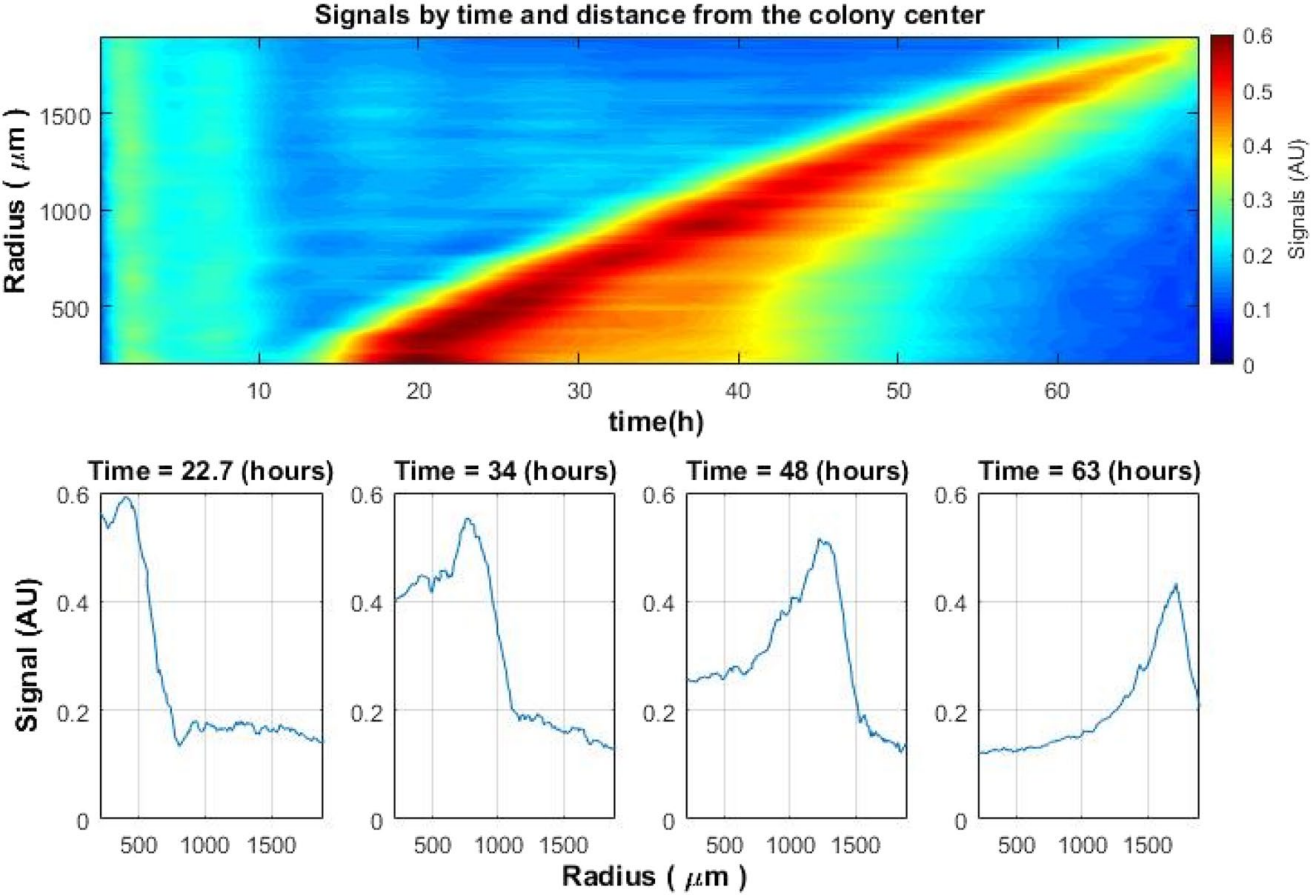
Envelopes of the averaged signals as a function of distance from colony center and time (top) and the peak of activity as a function of the distance from the center of the colony for different times (bottom) of *S. aureus*. Similar spatiotemporal behavior of the signal was observed also within colonies of *E. coli* and *V. natriegens.*

Taking the points corresponding to the distance from the center of the colony according to the curve described in Figs. 6 and 7, the coordinates in space (x, y) for each “ring” is obtained. A smoothed envelope function of the signal is placed at each such point in space (Fig. 5). Thus, many signals will correspond to each specific radius (the distance from the center of the colony to the maximum activity point). In an ideal situation, all signals within the same “ring” (at the same distance from the colony center) would be the same. However, the growth of a bacterial colony is affected by various conditions mentioned above so these signals are slightly different. Therefore, it is necessary to do some averaging of all signals for a given radius, so that each radius receives one signal that characterizes it. Such averaging was performed adopting the truncated average method^16^, where the truncation was performed not in amplitude, but in the time of the activity peak:

8 $$\overline{Env\left(D\right)}=\frac{1}{n-{k}_{b}-{k}_{f}}\left[Env\left({k}_{b}+1\right),...,Env\left(L-{k}_{f}\right)\right],$$

where L is the number of envelope signals at a given distance, ${k}_{b}$ and ${k}_{f}$—the number of envelope signals that were truncated from each side. That is, those signals whose peak of activity in the given radius differed greatly from the general trend (premature or delayed) were not considered. The obtained averaged signals were mapped as a function of distance from the center and time (Fig. 8, top image).

By selecting several points in time on the map, it is possible to observe how the peak of activity will look as a function of the distance from the center of the colony. The width of the peak is proportional to the width of the activity “ring”, and its change in time is shown in the bottom graph of Fig. 8.

### Spatial width of the activity zones

**Figure 9:**
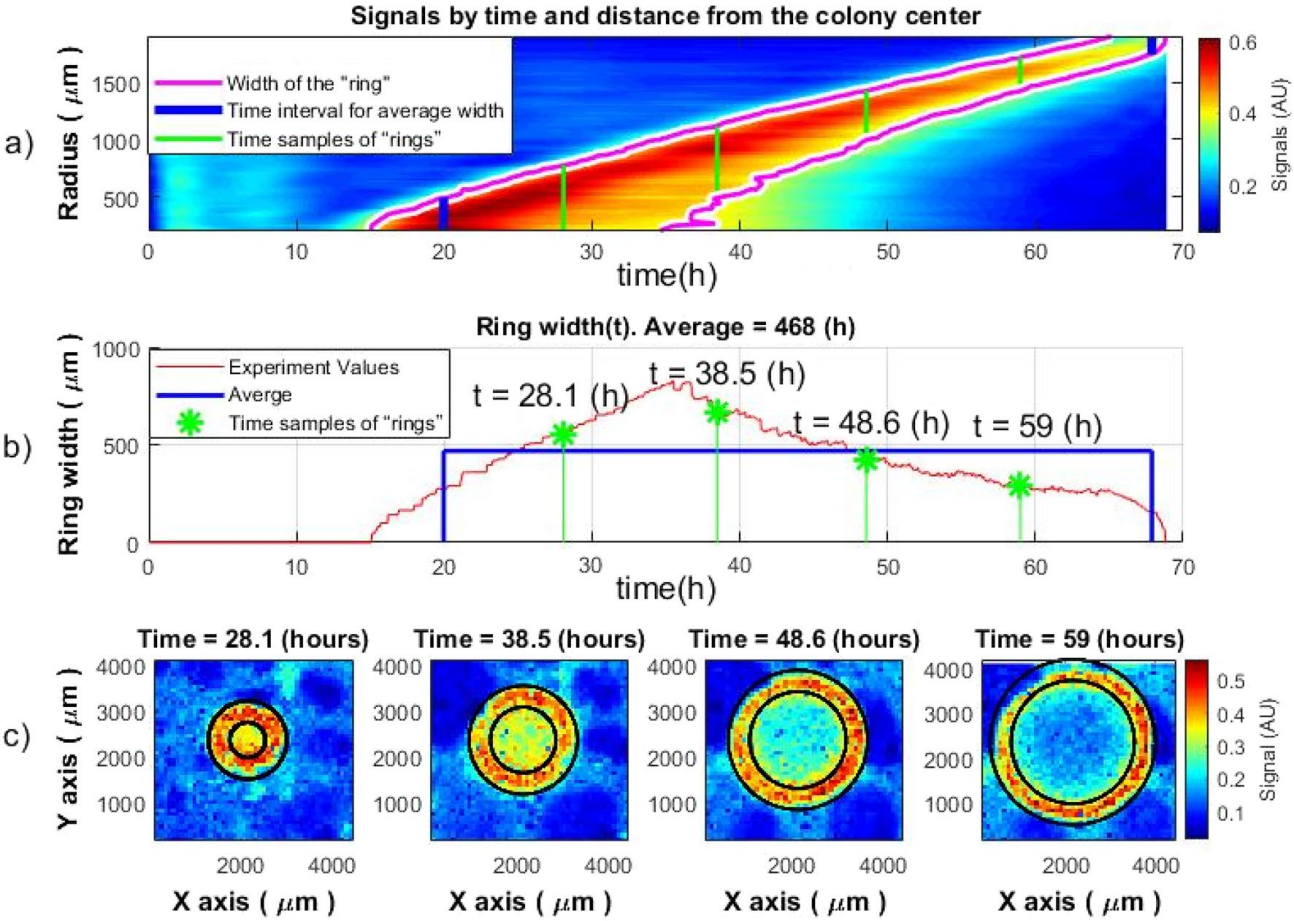
Determination of the width of the activity “ring” for the colony of *S. aureus*: (**a**) signal values and determined width of the activity ring (purple line), (**b**) the obtained width of the activity “ring”, (**c**) comparison of the obtained width of the activity “ring” (black lines) for one colony at different times. Similar spatiotemporal behavior of the signal was observed also within colonies of *E. coli* and *V. natriegens.*

The spatial width of the signal at different times can be observed in Fig. 8. The signal width corresponds to the decrease from the peak value to a certain value. If there were no noise, or unwanted signals, or any other factors, the criterion would be simple: reducing the signal level or its power from the maximum value to zero. However, in real conditions, there should be some threshold when the signal is still distinguishable against the noise. The decrease in signal power from the maximum to 1/3 of the maximum value was chosen as a criterion. That is, it should be noted that the actual width is slightly larger than the received one. It is possible to find the width for each moment. This width will match the width of the “ring” at the corresponding time (see Fig. 9).

**Figure 10:**
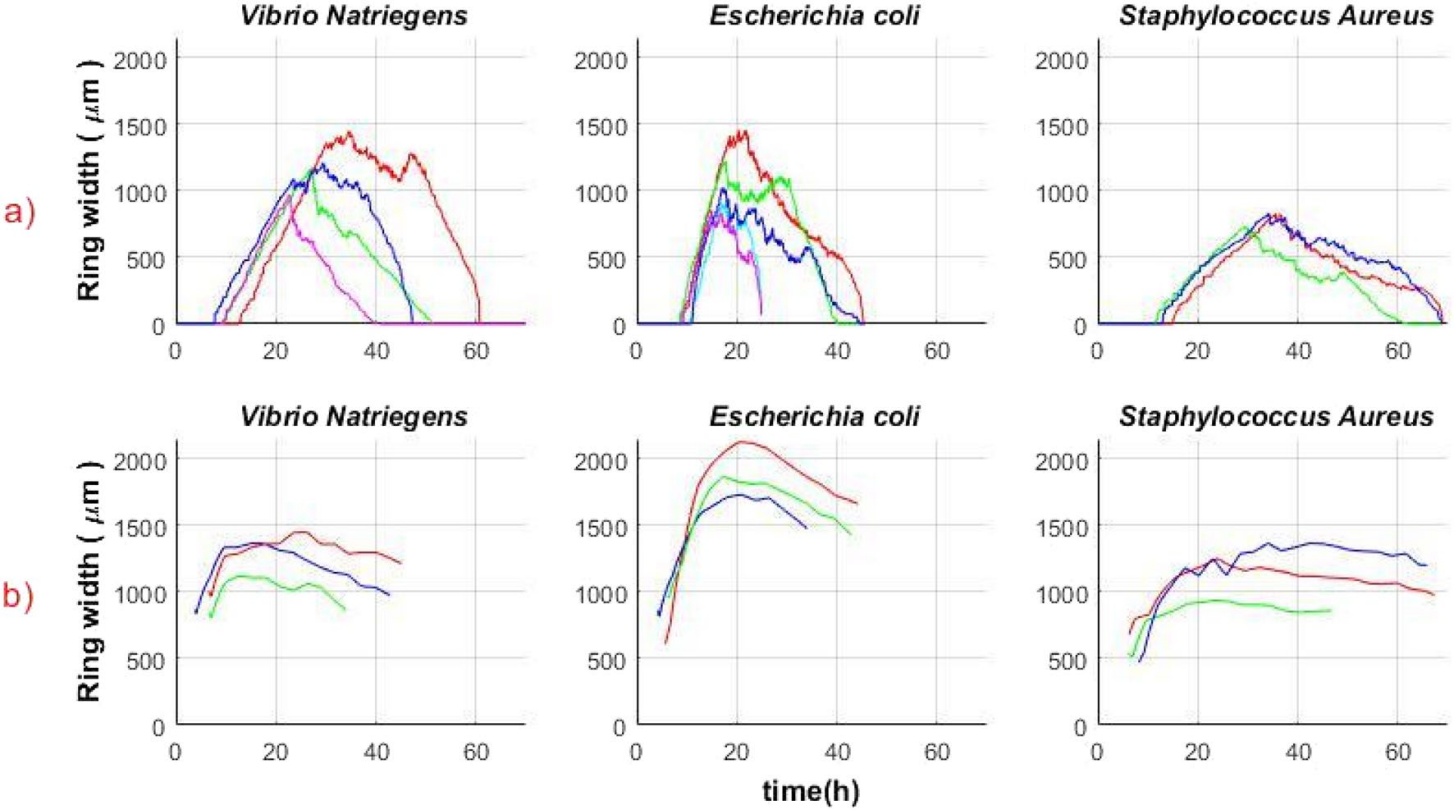
The width of activity “ring” as a function of time for different bacterial colonies; (**a**) width of activity “ring” determined by the speckle method; (**b**) calculated “ring” width using the Pirt model (Eq. 9). Parameters: alpha and r for the model are also obtained from speckle experiments. Each curve demonstrates the width of the activity “ring” over time of the one colony of a specific bacteria type.

From Fig. 9a,b it can be observed that although the change in ring width is small, it can be considered constant with some deviations. That is, from the time when it was possible to start observing the “ring” and until the end of the observation, when the “ring” disappeared, the width of the “ring” remained approximately unchanged (with reservations about the accuracy of measurement, random phenomena in the behavior of a colony of many microorganisms, noise, etc.). The behavior described for one selected colony was observed for all types of bacteria considered in this study. The width of the “ring” is different for each type of bacteria. Figure 10 shows the width of the “ring” for all types of bacterial colonies. The obtained results correspond to the mathematical model of the microbial colony growth introduced by John Pirt (“Pirt model”)^5^.

The Pirt study^5^ provides a formula for calculating the width of the active zone or the “ring”:

9 $$r=\frac{\omega \alpha }{2}t+{r}_{0},$$

where ω is the width of the active cell proliferation zone of the colony, r is the colony radius at time t, and ${r}_{0}$ is the radius at t = 0; α is the specific growth rate also known as (μ, h^−1^), which can be found from the Gompertz equation^8,17^. Thus, having the experimental data (the radius of the colony growth, the corresponding time, and μ), it is possible to calculate the width of the active zone or the width of the “ring” using Eq. (9). The results obtained in this study and the Pirt model are compared in Fig. 10.

The overall pattern of the ring width is unique and uniform for each bacterial species. However, activity ring width dynamics of each colony of a microbial species might be shifted in time. For longer growing colonies, the proliferation and thus speckle activity of the colony retains its activity longer. It may be assumed that this is related to the distribution of the colonies on the plate. Since the bacterial colonies on the agar plates were distributed randomly, their growth was affected by the neighboring microbial colonies. The growth of microbial colonies is strongly affected by neighboring colonies in proximity, so the more near-neighboring colonies a given colony has, the earlier its growth ceases due to the depletion of common nutritional resources of the media^18,19^.

The Pirt model predicts that the actively growing edge of the microbial colony might be of the similar width as measured by the speckle activity (compare Fig. 10*. V. natriegens* a and b), while it overestimates the width of the active edge of the microbial colony in the case of *E. coli* and *S. aureus.* It may be implied that the differences stem from the fact that growth rate α, h^−1^ in the Pirt equation is a constant, while in fact it changes (decreases) during colony growth^4^. Another aspect to consider is that in both cases experimental data were used for the calculations. However, as mentioned above, noise has influenced measurement accuracy.

## Discussion

Microbial colony growth on the agar media is a technique used in microbiology in research and routine tests to analyze microbial contamination of environmental, food or medical samples. Many mathematical models are used to describe microbial colony growth^4,5,18,20^. The width of the outmost activity zone—“the ring” of the colony—is often included in the mathematical models to simplify (linearize) the calculation of colony growth dynamics^5,21^. However, the experimental data on the actual size of the activity zone of the growing colony are scarce.

The Pirt model of microbial colony growth is among the first ones describing microbial colony radial growth over time. The model well aligns with real observations if there is no growth inhibition by the neighboring colonies and the size of the activity zone on the edge is constant. The Pirt model has been validated on the distantly growing macrocolonies arising from many cells^5^. However, in routine tests of environmental or medical samples, microcolonies growing at a random distance to the neighboring colonies are typically observed^22^. Additionally, due to the genomic or physiological make-up of microbial species, their colonies grow in different 3D shapes (dome-shaped, flat, etc.), which in turn might influence the width of the activity zone on the edge of the colony^23^.

In one of the recent models suggested by Warren et al.^24^, the orientation of the microbial cells (horizontal or vertical) together with the gradual nutrient depletion around the colony define the dynamics of the colony growth. *Warren* and colleagues in their simulations noticed the formation of a metabolically active “ring” structure around the colony, which is formed by cells having direct contact with the substrate, and where most proliferation activity takes place^24^.

In this (our) study, a laser speckle-based system was developed to record microbial colony growth together with a sub-pixel correlation analysis algorithm of laser speckle images, which allows discriminating and quantifying activity zones within the colony. The speckle signal is in good correlation with the colony growth rate and is dependent on the cell number per colony (see Figs. 3 and 4).

Certain scatter of the parameters recorded by speckle imaging (activity ring width, maximum migration distance from the center, microbial colony growth curves) is observed, which might be attributed to the changes of properties of the agar media, biological variability of the sample and/or accuracy of the measurements.

**Figure 11:**
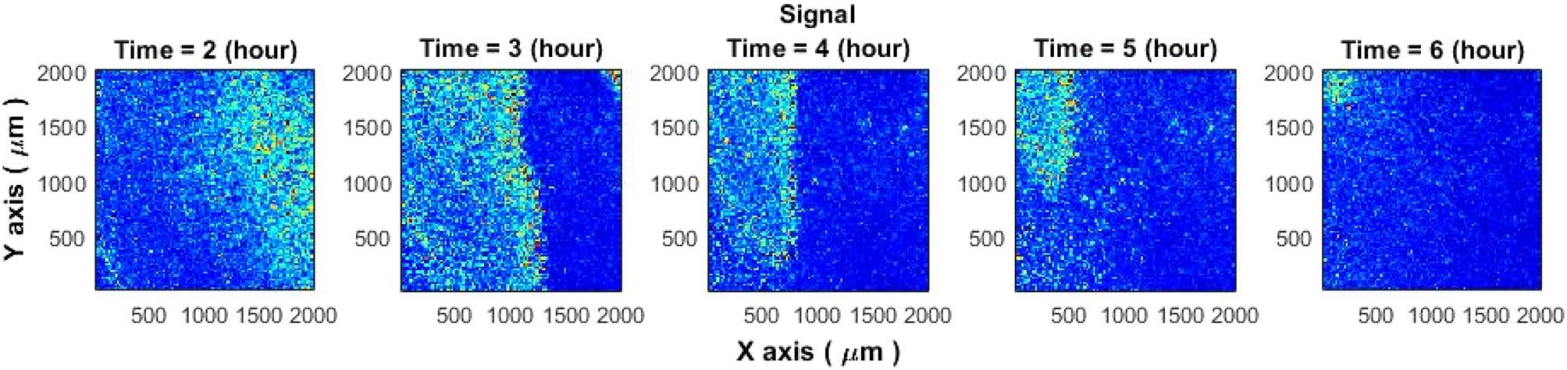
Speckle imaging of the drying agar (agar media without bacteria) in a Petri dish.

It is known that agar plates tend to lose water (dry out) during microbial cultivation^25^. Formation and strength of the speckle signal might be affected by the agar media dry out. According to our observations, in many experiments in the first 3–5 h, effects associated with the drying of the agar media might occur. However: (1) This effect spread throughout the Petri dish like a "wave" and then disappeared without causing additional effects. (2) The observed effect of "drying" occurred much earlier than the formation of "rings". Accordingly, even if drying appeared, this effect did not affect the formation of rings. An example of the drying effect is shown in Fig. 11. Besides, Sandle et al. observed larger weight loss at the very beginning (0–4 h) of the cultivation rather than at the end.

**Figure 12:**
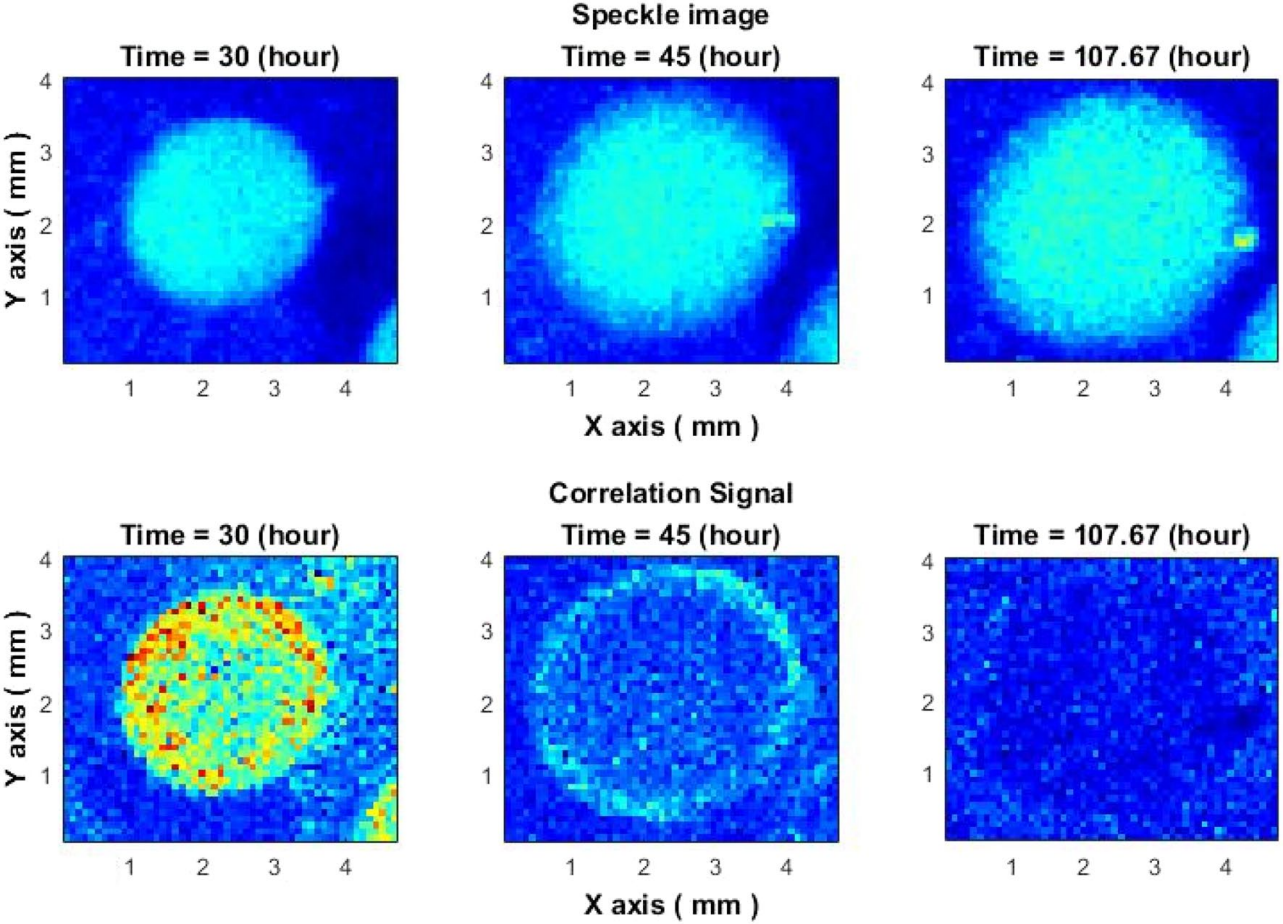
Activity ring signal ceases over time in aging colonies of *Vibrio natriegens*. Top: colony speckle images, Bottom: image after applying subpixel correlation algorithm, cease of activity ring.

The growth of the colonies on the agar media was similar after the inoculation-colonies tend to have the same initial growth rate (see Fig. 3) and their growth diverged from each other later on: colonies ceased to grow in different times. Since allocation of the colonies on the plate were random—with variable number of neighboring colonies, the depletion of the resources and subsequently growth cessation for every colony could occur in different times. Nevertheless, each colony exhibited an activity ring with characteristic migration away from the center and cessation over time when active growth diminishes (see Fig. 12).

When a colony has ceased to be active, it is still visible on raw speckle images (Fig. 12 (top)) but ceases to demonstrate a ring effect (Fig. 12 (bottom)). Thus, it is concluded, that "rings" are not random phenomena related to agar desiccation, nor artifact of colony edge, but a signal of microbial activity, which would disappear when the colony stops growing.

The proposed speckle technique allows identifying and recording the dynamics of the width of the activity zone throughout the growth of the micro and macro colony. In the future, colony growth recording using the laser speckle might become an attractive, non-invasive tool for quantifying growth dynamics and proliferation activity with sub-colony resolution.

## Conclusions

The speckle imaging method provides more insights into the microbial activity within the colonies than standard imaging series under white light.

It has been demonstrated that the laser speckle imaging technique is a truly non-invasive method, allowing to monitor uninterrupted microbial colony growth (the growth rate of the colonies under speckle system was statistically indistinguishable from the microbial growth in reference conditions). After applying the subpixel correlation algorithm (“Algorithm of the speckle image conversion into time signals”), the maximum appearance time of the speckle signal was proportional to cell number in the colony (Fig. 4).

Sensitive correlation subpixel analysis revealed that distinct activity zone within the colony exist that migrate from the center to the edges during the growth of the colony. We observed a similar pattern of activity zone (''ring'') migration in the colonies of three different microbial species. Moreover, we observed the presence of the ring in every actively growing colony (see Supplementary Fig. S1). The migration speed of the “ring” varied across different microbial species and thus reflected their different growth rates typical for each of these species.

Speckle imaging is promising, powerful technology which for the first time has helped to visualize activity zones within the microbial colony. Previously the presence of these zones were predicted mathematically. Current study also suggests the potential ways to further develop speckle imaging technology for microbial colony studies: to increase the sensitivity of optical and electronic systems for speckle detection, to minimize noise, to seek the options for multiplexing the technology. It would allow recording microbial proliferation activity as early as possible, as well as detecting slowly growing colonies, and identify microbial activity within the colony which makes the proposed technique suitable for many direct applications in research and development, medicine, and epidemiology.

## Supplementary Information

Supplementary Information.

## Data Availability

The datasets used and/or analysed during the current study available from the corresponding author on reasonable request.
