# Supplementary material for: Use of the speckle imaging sub-pixel correlation analysis in revealing a mechanism of microbial colony growth
Source: Sci Rep. 2023 Feb 14;13:2613. doi: 10.1038/s41598-023-29809-0 (PMC9929235; doi:10.1038/s41598-023-29809-0)
Supplement: Supplementary file 1 — Supplementary Information. [file 41598_2023_29809_MOESM1_ESM.docx]

Supplementary material *table 1*

| radial growth rate in speckle recording system, um * h^-1^ | | | |
| --- | --- | --- | --- |
|  | *Escherichia coli* | *Staphylococcus aureus* | *Vibrio natriegens* |
| 8_17 h | 145.65 | 45.69 | 65.30 |
| 8_17 h | 116.57 | 37.00 | 61.55 |
| 8_17 h | 130.07 | 46.25 | 68.50 |
|  |  |  |  |
|  |  |  |  |
| radial growth rate in scanner, um * h^-1^ | | | |
|  | *Escherichia coli* | *Staphylococcus aureus* | *Vibrio natriegens* |
| 8_17 h | 125,52 | 47,57 | 70.47 |
| 8_17 h | 139,45 | 52,25 | 69.79 |
| 8_17 h | 136,32 | 51,10 | 73.67 |
| 8_17 h | 142,77 | 47,10 | 70.40 |
| 8_17 h | 138,96 | 47,33 | 72.76 |
| 8_17 h | 140,37 |  | 71.42 |
| 8_17 h | 143,27 |  |  |
| 8_17 h | 152,71 |  |  |
| 8_17 h | 155,14 |  |  |
| 8_17 h | 130,56 |  |  |

The radial growth rate was measured during exponential growth phase (between 8 and 17 h)

Calculated p values after two tailed, unequal variance t-test

|  | *E. coli* speckle vs scanner | *S. aureus* speckle vs scanner | *V. natriegens* speckle vs scanner |
| --- | --- | --- | --- |
| experimental  p value | 0.57 | 0.17 | 0.07 |

None of experimental p values are smaller than 0.05, thus - the difference is statistically insignificant.

Supplementary material *Figure S 1*

*
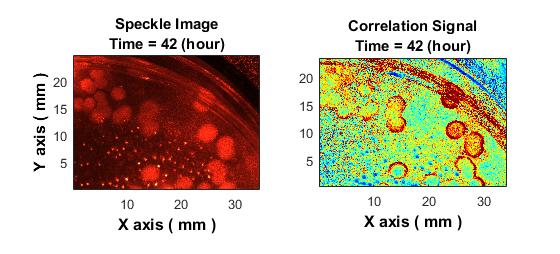
*

figure S1.  Demonstration of the ring effect on a part of a Petri dish with  about 25  bacterial colonies
